# Genome-Wide Transcriptome Analysis of CD36 Overexpression in HepG2.2.15 Cells to Explore Its Regulatory Role in Metabolism and the Hepatitis B Virus Life Cycle

**DOI:** 10.1371/journal.pone.0164787

**Published:** 2016-10-17

**Authors:** Jian Huang, Lei Zhao, Ping Yang, Zhen Chen, Ni Tang, Xiong Z. Ruan, Yaxi Chen

**Affiliations:** 1 Centre for Lipid Research & Key Laboratory of Molecular Biology for Infectious Diseases (Ministry of Education), Institute for Viral Hepatitis, Department of Infectious Diseases, the Second Affiliated Hospital, Chongqing Medical University, Chongqing, China; 2 John Moorhead Research Laboratory, Centre for Nephrology, University College London Medical School, Royal Free Campus, University College London, London, United Kingdom; Temple University School of Medicine, UNITED STATES

## Abstract

Hepatitis B virus (HBV) is a hepatocyte-specific DNA virus whose gene expression and replication are closely associated with hepatic metabolic processes. Thus, a potential anti-viral strategy is to target the host metabolic factors necessary for HBV gene expression and replication. Recent studies revealed that fatty acid translocase CD36 is involved in the replication, assembly, storage, and secretion of certain viruses, such as hepatitis C virus (HCV) and human immunodeficiency virus (HIV). However, the relationship between CD36 and the HBV life cycle remains unclear. Here, we showed, for the first time, that increased CD36 expression enhances HBV replication in HepG2.2.15 cells. To understand the underlying molecular basis, we performed genome-wide sequencing of the mRNA from HepG2.2.15-CD36 overexpression (CD36OE) cells and HepG2.2.15-vector cells using RNA Sequencing (RNA-seq) technology to analyze the differential transcriptomic profile. Our results identified 141 differentially expressed genes (DEGs) related to CD36 overexpression, including 79 upregulated genes and 62 downregulated genes. Gene ontology and KEGG pathway analysis revealed that some of the DEGs were involved in various metabolic processes and the HBV life cycle. The reliability of the RNA-Seq data was confirmed by qPCR analysis. Our findings provide clues to build a link between CD36, host metabolism and the HBV life cycle and identified areas that require further investigation.

## Introduction

Hepatitis B virus (HBV) infection is a major cause of morbidity and mortality around the world because it enhances the development of hepatitis, cirrhosis and hepatocellular carcinoma [1]. HBV is a small liver-specific targeting DNA virus. It consists of partially double-stranded DNA that can be bound with a variety of cellular transcription factors to regulate viral gene expression, some of which are activators of hepatic genes related to metabolism, for example, glucose and lipid synthesis and consumption[2].Therefore, HBV is designated as a “metabolovirus” due to the unique interaction between the virus and host metabolic processes in the liver [3]. A potential antiviral strategy is to target the host factors necessary for HBV gene expression.

Cluster of differentiation 36 (CD36), also known as fatty acid translocase, is a transmembrane glycoprotein that is widely expressed in various tissues, including the liver, heart, muscle and adipose tissue[4]. It is mainly involved in the metabolism of fatty acids, including the uptake of long-chain fatty acids (LCFA), accumulation of lipids and some metabolic dysfunctions under the conditions of excessive fatty acid supply[5]. In addition to LCFA, CD36 can recognize lipid ligands, such as oxidized phospholipids and cholesterol, and its binding behavior can initiate signal transduction in many cells. Because of these signal transduction capabilities, CD36 is involved in inflammation, immune response, angiogenesis, atherogenesis and thrombosis[6]. We do not yet fully understand the roles of CD36 in human liver diseases, but significant progress has been recently made. A study found that CD36 contributed to HCV replication by facilitating viral attachment on the host cell membrane and CD36 inhibitors blocked HCV replication in vitro [7]. Recent studies have revealed that CD36 silencing in human immunodeficiency virus (HIV)-infected macrophages showed decreased amount of virions released, which could also be inhibited by soluble anti-CD36 antibodies. The transmission of virus to CD4+ T cells was also influenced [8]. These results suggest that CD36 is required for the HCV and HIV life cycle. Thus, CD36 could be a potential drug target against HCV and HIV.

Many studies have found that CD36 is involved in lipid metabolism disorder, such as metabolic syndrome (MS), nonalcoholic fatty liver disease (NAFLD) [9, 10]. A population-based study demonstrated that patients with MS had increased HBV DNA loads compared with those without MS [11]. However, whether CD36 is involved in HBV replication is still unclear. We preliminarily demonstrated that the expression of CD36 was increased in HBV-expressing cell line, suggesting a possible function of CD36 in modulating HBV replication. Here, the HepG2.2.15 cell line, which supports HBV stable replication, protein expression, and virion assembly and secretion [12], was employed as a model to further investigate the relationship among CD36, the HBV life cycle and host metabolism. We first showed that CD36 overexpression could enhance HBV replication in HepG2.2.15 cells. To uncover the potential role of CD36 in the HBV life cycle, a comparative transcription analysis was performed in HepG2.2.15-CD36 overexpression (CD36OE) and HepG2.2.15-vector stable cell lines using RNA-seq technology to detect the differential transcriptomic profile. The goal of this study was to identify differentially expressed genes (DEGs) between HepG2.2.15-CD36OE and HepG2.2.15-vector cells to improve the understanding of the molecular mechanism associated with increased CD36 expression in HBV replication cell lines. The main function of the DEGs was analyzed by gene ontology and pathway analysis. Then, gene co-expression networks were constructed to identify the interactions among the DEGs. In addition, real-time PCR was performed to validate the expression changes of the DEGs involved in CD36 overexpression.

## Methods

### Cells culture and construction

Human hepatocellular carcinoma cell line HepG2 and HepG2.2.15 cells (stably HBV transfected HepG2 cells, as well as expression of virus related proteins) (preserved in Chongqing Key Laboratory of Molecular Biology for Infectious Diseases) were maintained with modified Eagle’s medium (MEM) containing 10% fetal calf serum, and HepG2.2.15 cells required additional 500 μg G418/mL. To generate HepG2.2.15 cells that could stably overexpress CD36, human CD36 cDNA was cloned into the GV341 plasmid. Next, a lentivirus-CD36 overexpression system was constructed, packaged, and purified by GeneChem. CD36-overexpression-lentivirus and vector-lentivirus were transfected into HepG2.2.15 cells and were designated as HepG2.2.15-CD36OE and HepG2.2.15-vector cells, respectively. Subsequently, puromycin (2 μg/mL) was used to select stably transfected cell lines.

### Western blotting

To identify CD36 expression in HepG2, HepG2.2.15, HepG2.2.15-CD36OE and HepG2.2.15-vector cells, cells were lysed using RIPA containing a protease inhibitor cocktail (Roche, Germany), and the protein concentration was measured and normalized for western blotting. Proteins (50 μg) were separated by 8% SDS-PAGE gradient gels and transferred onto PVDF membranes. After blocking with 3% BSA, membranes were incubated with antibody against human CD36 (Abcam, UK) at 4°C overnight. The membranes were then incubated with the corresponding secondary peroxidase-coupled antibody. Finally, the detection procedure was performed using an ECL Advance Western Blotting Detection kit (Millipore, USA). Densitometric analysis was performed using Image J software (National Institutes of Health,USA). An anti-β-actin antibody was used as a loading control.

### Southern blot analysis

Core particles associated with HBV DNA replicative intermediates were extracted from HepG2.2.15-CD36OE and HepG2.2.15-vector cells after 96 hours, according to the previously described method[13]. HBV DNA samples were separated by electrophoresis in 1% agarose gel and transferred onto nylon membranes. UV cross-linking, prehybridization, and hybridization were performed according to the protocol from the manufacturer of the DIG-High Prime DNA Labeling and Detection Starter Kit I (Roche, Germany). The signal was detected by exposure on an X-ray film.

### Quantification and differential expression analysis of transcripts

Total RNA was extracted using TRIzol reagent (Invitrogen, USA) according to the protocol provided by the manufacturer, after which the concentration was measured by a NanoDrop 2000 (Thermo Scientific, USA) with the quality assessed by an Agilent 2200 (Agilent, USA). An RNA sequencing library of each sample was prepared using the Ion Total RNA-Seq Kit v2, in accordance with the protocol provided by the manufacturer (Life technologies, USA). After library construction, each library preparation was sequenced using the Ion PI^™^ chip, and the raw reads were generated. After filtering, the clean reads were used to map to the reference genome using Misplacing software[14]. The expression level of each gene was normalized by the reads per kilobase per million (RPKM) [15]. To identify DEGs, the DEseq package was used to filter the DEGs for the HepG2.2.15-CD36OE and HepG2.2.15-vector groups[16]. After the statistical analysis, we screened the DEGs with a fold change > 1.5 or fold change < 0.667, and the false discovery rate (FDR) threshold was FDR < 0.05.

### Gene ontology and pathway analysis

To identify the main function of the DEGs, gene ontology (GO) analysis was performed using the GO-TermFinder tool [17]. Significantly enriched GO terms were detected according to a p-value < 0.05. Moreover, to identify the significantly enriched pathways, pathway annotation was performed according to the KEGG database [18]. The significantly enriched pathways were computed on the basis of the Fisher test of the hyper geometric distribution at a p-value < 0.05.

### Co-expression network analysis

All DEGs were used to construct gene co-expression networks on the basis of the normalized signal intensity of specific genes. The Pearson’s correlation of each pair of genes was computed, and the significantly correlated pairs were selected to construct the co-expression network [19]. The centrality degree of a gene was assigned in terms of the number of links that one node has with another [20, 21]. The centrality degree represents the centrality of genes in the network and can reflect the relative importance of the gene. In addition, the k-cores were adopted so that all nodes are connected to at least k other genes in the subnetwork [22]. Within the networks analysis, firstly, the DEGs with the highest difference centrality degree (absolute value≥6) were selected; secondly, among above screening genes, the genes with the highest difference k-core scoring (absolute value≥6) were recognized as potential core regulatory genes.

### Quantitative real-time PCR validation

To identify CD36 overexpression, the total RNA of HepG2.2.15-CD36OE and HepG2.2.15-vector cells was extracted and reverse-transcribed to cDNA for quantification by real-time PCR. To validate the RNA-Seq results, cDNA was synthesized using 1 μg of the original RNA sample by reverse transcription according to the protocol, and these cDNA templates were used for further quantitative PCR analysis. Quantitative real-time PCR (qPCR) using the SYBR Green method was used to detect the gene expression level. β-actin was chosen as the reference gene, and the expression levels were computed according to the 2^−ΔΔCt^ values. The primer sequences are provided in S1 Table.

## Results

### CD36 overexpression enhanced HBV replication in HepG2.2.15 cells

**Fig 1:**
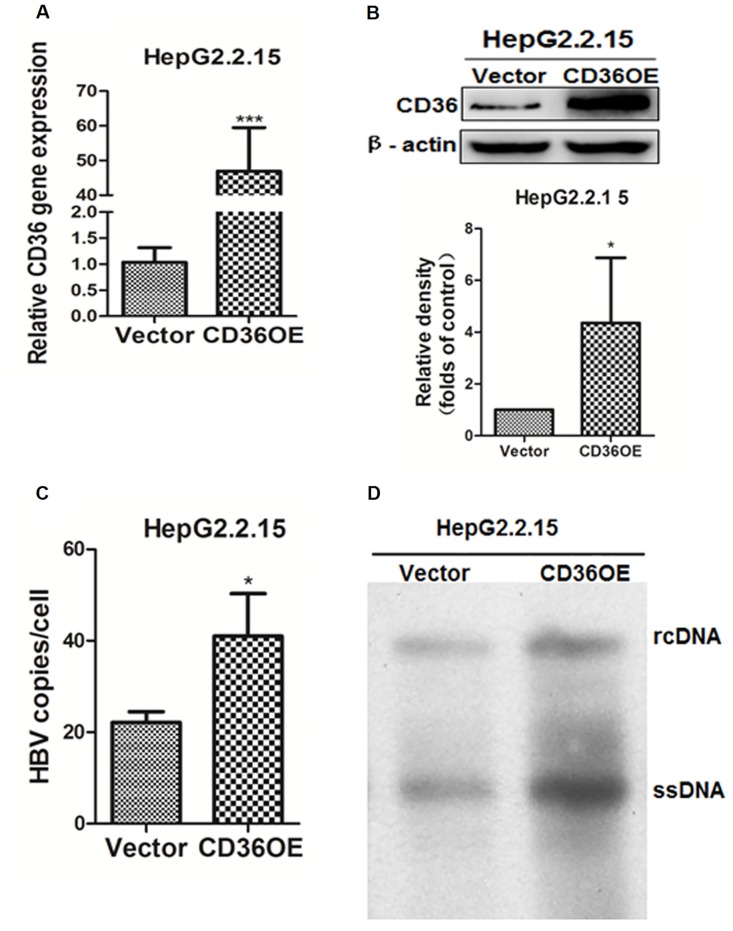
CD36 overexpression enhanced HBV replication in HepG2.2.15 cells. (A and B) The mRNA and protein levels of CD36 were analyzed in HepG2.2.15 cells with the stable overexpression of CD36. (C and D) HBV replication was enhanced in HepG2.2.15 cells with stable overexpression of CD36 compared with the vector control. Core particles associated with HBV intermediates were extracted after 4 days and were identified by qPCR (C) and Southern blotting (D). The values are presented as the means± SD (n≥3). Comparisons among groups were determined with independent-sample t-test using the SPSS software (Version 11.5). Significant differences are considered at *P<0.05 and *** p < 0.0001 versus control group.

To identify a potential relationship between HBV replication and CD36, we firstly examined the expression of CD36 in HepG2.2.15 and its parental HepG2 cells. As compared with HepG2 cells, the protein level of CD36 was significantly upregulated in HepG2.2.15 cells (S1 Fig). The results revealed that CD36 was significantly upregulated by HBV replication, suggesting that CD36 might play a role in HBV replication. To further understand the relationship between CD36 and HBV, we constructed CD36 overexpression cell lines in HepG2.2.15 cells using the lentivirus-CD36 overexpression system. Our results showed that the CD36 mRNA and protein levels were significantly higher in the HepG2.2.15-CD36OE group than in the HepG2.2.15-Vector group (Fig 1A and 1B), suggesting that the cell model of CD36 overexpression was successfully established. Next, HBV DNA replicative intermediates were detected by both qPCR and Southern blotting. The data showed that upregulation of CD36 resulted in enhanced levels of HBV DNA in HepG2.2.15 cells (Fig 1C and 1D).

### RNA-seq of the HepG2.2.15-CD36OE and HepG2.2.15-vector cell transcriptome

To better understand the molecular changes in response to CD36 overexpression in HepG2.2.15 cells, three cDNA libraries from HepG2.2.15-vector cells (Vector-1, Vector-2, and Vector-3) and HepG2.2.15-CD36OE cells (CD36OE-1, CD36OE-2, and CD36OE-3) were constructed. After performing quality control, an Ion Proton^TM^ Sequencer was used to sequence the cDNA libraries. High-throughput sequencing yielded 15.30, 14.68, and 17.46 and 13.74, 14.37, and 18.87 million total raw reads from HepG2.2.15-vector cells and HepG2.2.15-CD36OE cells (S2 Table), respectively. After filtering the raw reads, 14.34, 13.71, and 16.13 and 12.99, 13.54, and 17.50 million clean reads were obtained. More than 92% of the filtered reads were used for downstream analysis.

To further analyze the gene expression profile, the clean reads in the six libraries were compared to the human reference genome using the MapSplice tool. The results showed that the proportions of clean reads (90.9%, 91.3%, and 90.7% and 93.4%, 92%, and 90.7% in the HepG2.2.15-vector and HepG2.2.15-CD36OE cell libraries, respectively) matched the human reference genome (Table 1). Moreover, some of these clean reads were uniquely mapped to the reference genome: 12.49 (87.1%), 12.00 (87.6%), and 14.07 (87.2%) million reads in the HepG2.2.15-vector libraries and 11.66 (89.8%), 11.95 (88.2%), and 15.22 (86.9%) million reads in the HepG2.2.15-CD36OE libraries.

**Table 1:** Summary of RNA-seq data mapped to the reference genome.

Sample name	Vector-1	Vector-2	Vector-3	CD36OE-1	CD36OE-1	CD36OE-1
Allreads	14335354	13706017	16130270	12993601	13543156	17500321
UnMapped	1304643	1198305	1495013	860501	1083308	1631453
Mapped	13030711	12507712	14635257	12133100	12459848	15868868
MappedRate	90.9%	91.3%	90.7%	93.4%	92.0%	90.7%
UniqueMapped	12489663	12004214	14067500	11661802	11947415	15216321
UniqueMappedRate	87.1%	87.6%	87.2%	89.8%	88.2%	86.9%
RepeatMapped	541048	503498	567757	471298	512433	652547
JunctionAllMapped	4345060	4295847	4926564	4381305	4262410	5307358
JunctionUniqueMapped	4338301	4289175	4919678	4369591	4253552	5299893
AllBase	2183679165	2083371812	2481865449	2080596567	2151759352	2721359004
UnMappedBase	156007543	141200940	176569476	103653653	130614823	188418418
MappedBase	2027671622	1942170872	2305295973	1976942914	2021144529	2532940586
UniqueMappedBase	1949440314	1870217136	2222958444	1906932384	1944226993	2438795965
RepeatMappedBase	78231308	71953736	82337529	70010530	76917536	94144621

### Analysis of gene differential expression

To identify the DEGs, the number of unique clean reads for each gene was computed and normalized to RPKM. The RPKM distribution showed there were few different genes in the same groups (S2 Fig). The criteria of fold change > 1.5 or fold change < 0.667 in expression and FDR < 0.05 were chose to screen the significantly upregulated or downregulated genes. A total of 141 DEGs were identified according to these criteria, including 79 upregulated genes and 62 downregulated genes (S3 Table). Some genes associated with HIV were also found in this analysis, including apolipoprotein B mRNA editing enzyme catalytic subunit 3B (*APOBEC3B*), C-C motif chemokine ligand 20(*CCL20*), PDZ domain containing 8(*PDZD8*), and beta-site APP-cleaving enzyme 2(*BACE2*). Together, our results demonstrated that there were distinct gene regulation patterns between the HepG2.2.15-CD36OE group and the HepG2.2.15-vector group, suggesting that CD36 overexpression can produce unique gene profiles, which may be related to the HBV life cycle.

### Functional annotation of differentially expressed genes

**Fig 2:**
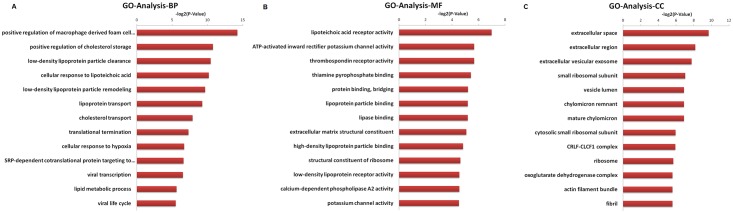
Gene Ontology analysis of the differentially expressed genes. The GO categories include biological processes, molecular functions and cellular components, respectively.

**Fig 3:**
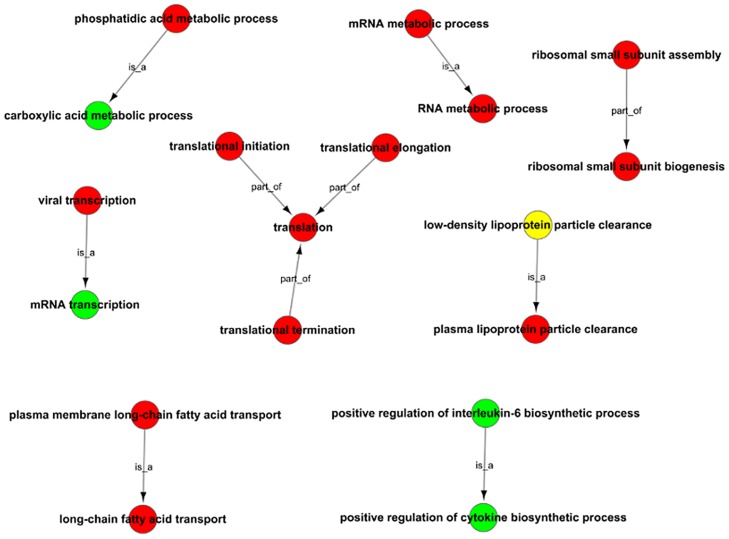
GO trees of the GO terms. Red indicates the GO terms of the upregulated genes, green indicates the GO terms of the downregulated genes, and yellow indicates the GO terms from both upregulated and downregulated genes.

To understand the functions of the DEGs, GO analysis was carried out. According to the GO terms, 66, 74, and 64 DEGs were classified into biological processes, molecular functions and cellular components, respectively. Compared to the HepG2.2.15-vector group, the highly enriched GO terms of biological processes in the HepG2.2.15-CD36OE group were related to positive regulation of macrophage-derived foam cell differentiation, positive regulation of cholesterol storage, low-density lipoprotein particle clearance, cellular response to lipoteichoic acid, and low-density lipoprotein particle remodeling (Fig 2A). In the category of molecular function, the most significant subcategories were lipoteichoic acid receptor activity, ATP-activated inward rectifier potassium channel activity, thrombospondin receptor activity, thiamine pyrophosphate binding, and protein binding (Fig 2B). The main subcategories of the cellular component were extracellular space, extracellular region, extracellular vesicular exosome, and small ribosomal subunit (Fig 2C). These results showed that CD36 overexpression was mainly involved in the regulation of lipid metabolic process, lipoprotein transport, translation and viral process. Three genes of ribosomal protein S25(*RPS25*), ribosomal protein L10(*RPL10*) and ribosomal protein S28(*RPS28*) contribute to the GO terms of viral transcription and the viral life cycle.Furthermore, to understand the functional relationships of the GO terms, GO trees were constructed (Fig 3). As shown in Fig 3, the genes in the HepG2.2.15-CD36OE groups were highly enriched in categories related to long-chain fatty acid transport, plasma lipoprotein particle clearance carboxylic metabolic processes, translation, RNA metabolic processes, mRNA transcription, positive regulation of cytokine biosynthetic process and ribosomal small subunit biogenesis compared to the HepG2.2.15-vector group. These GO enrichment categories provide clues about the role of CD36 in HBV replication cell lines, which will facilitate screening of the genes that contribute to these categories.

KEGG enrichment analysis was performed to further investigate the potential function of these DEGs. These 141 DEGs were mapped to 56 pathways. Ultimately, five statistically significant enrichment pathways were identified according to the hypergeometric test (p < 0.05). These pathways were involved in fat digestion and absorption, tryptophan metabolism, arachidonic acid metabolism, ribosomes, and gastric acid secretion (Table 2).

**Table 2:** The significantly statistical enrichment pathways of differentially expressed genes.

Pathway ID	Pathway Term	Pathway gene	P-Value	Enrichment	(-log2P)
PATH:04975	Fat digestion and absorption	APOB,PLA2G2A	0.0007104	20.075099	10.459042
PATH:00380	Tryptophan metabolism	OGDHL,DDC	0.0102091	14.658009	6.6139949
PATH:03010	Ribosome	RPS28, RPS25,RPL10	0.0128876	6.840404	6.2778757
PATH:00590	Arachidonic acid metabolism	GPX2, PLA2G2A	0.0244863	9.0534759	5.3518817
PATH:04971	Gastric acid secretion	KCNJ10, KCNK10	0.0291768	8.2084848	5.0990353

### Co-expressed genes and their networks

**Fig 4:**
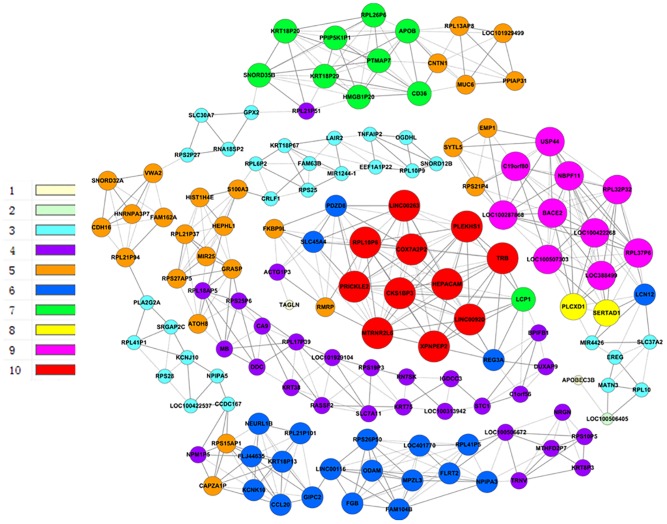
Gene co-expression network analysis for the HepG2.2.15-vector group. The lines represent regulatory relationships: solid lines indicate positive correlations; dashed lines indicate negative correlations. The volume of the node represents the degree centrality of co-expression, the color represents the “k-core” scores.

**Fig 5:**
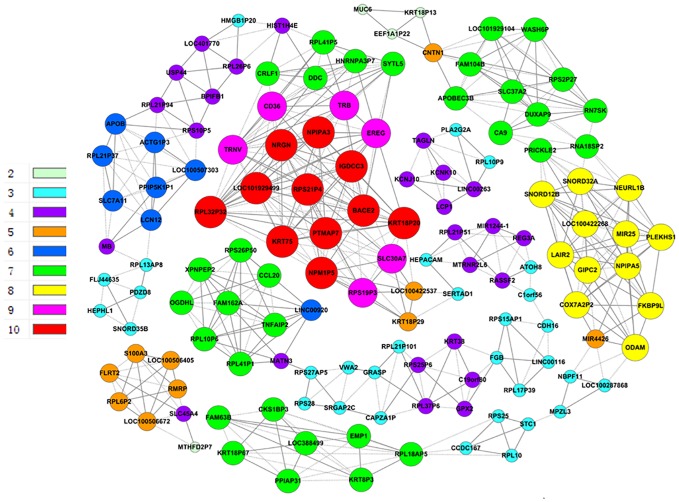
Gene co-expression network analysis for the HepG2.2.15-CD36OE group. The lines represent regulatory relationships: solid lines indicate positive correlations; dashed lines indicate negative correlations. The volume of the node represents the degree centrality of co-expression, the color represents the “k-core” scores.

Furthermore, we constructed general co-expression networks of DEGs to identify the core genes accompanying CD36 overexpression. In the HepG2.2.15-vector group, the co-expression network had a total of 496 connections between genes (Fig 4). Within this co-expression network, 254 pairs presented as positive and 242 pairs presented as negative. In the HepG2.2.15-CD36OE group, the co-expression network showed a total of 516 connections between genes (Fig 5). Within this co-expression network, 269 pairs presented as positive and 247 pairs presented as negative. The networks showed that there were markedly different patterns of gene co-expression in the two groups. Total 11 genes were selected as potential core regulatory genes according to the highest difference centrality degree and highest difference k-core scoring (Table 3). Furthermore, according to GO analysis, we found that the functions of some core genes were related to deoxycytidine deaminase activity, calmodulin binding, epidermal growth factor receptor binding, structural molecule activity, cation transmembrane transporter activity.

**Table 3:** Core genes between the co-expression networks of HepG2.2.15-CD36OE and HepG2.2.15-vector.

Gene symbol	Centrality degree in CD36OE group	Centrality degree in vector group	K-core in CD36OE group	K-core in vector group	Degree Difference	K-core Difference
IGDCC3	15	4	10	4	11	6
NRGN	16	5	10	4	11	6
EREG	15	5	9	3	10	6
KRT75	16	7	10	4	9	6
SLC30A7	11	3	9	3	8	6
APOBEC3B	9	1	7	1	8	6
LINC00263	6	12	4	10	-6	-6
LOC100287868	4	12	3	9	-8	-6
NBPF11	4	12	3	9	-8	-6
MTRNR2L6	5	14	4	10	-9	-6
HEPACAM	4	12	3	10	-8	-7

### Confirmation of differentially expressed genes by quantitative real-time PCR

**Fig 6:**
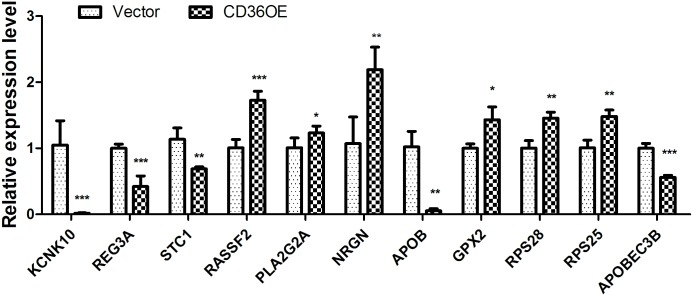
Validation of the RNA-Seq results using quantitative real-time PCR. The values are presented as the means±SD (n = 4). Comparisons among groups were determined with independent-sample t-test using the SPSS software (Version 11.5). Significant differences are considered at *P<0.05, ** p < 0.001 and *** p < 0.0001 versus control group.β-actin was used as the reference gene.

To validate the expression levels of the DEGs, 11 DEGs were randomly selected for validation by qPCR. As shown in Fig 6, the qPCR results of the DEGs were in agreement with those of the RNA-Seq analysis, suggesting the RNA-Seq data used in the present study was reliable and accurate.

## Discussion

In this study, we first reported that the CD36 protein levels were significantly higher in the HepG2.2.15 cells than in its parental HepG2 cells. Further results demonstrated that CD36 overexpression could significantly enhance HBV replication in HepG2.2.15 cell lines. To reveal the potential molecular mechanism, we performed genome-wide analysis of mRNAs from HepG2.2.15-vector cells and HepG2.2.15-CD36OE cells to detect DEGs involved in metabolism and the HBV life cycle. To our knowledge, this is the first genome-wide analysis of mRNAs to screen DEGs that were induced by increasing CD36 expression. Therefore, we propose that these data will serve as a powerful tool and resource for future research related to the effects of CD36 on host metabolism and the HBV life cycle.

The RNA-Seq results identified 141 DEGs. Among these DEGs, some virus-related genes were differentially regulated between the two cell lines, including *APOBEC3B*, *CCL20*, *PDZD8*, and *BACE2*, which are known to be associated with the HIV-1 virus life cycle or to interact with HIV-1 proteins[23–26]. Whether these DEGs were participants in the HBV life cycle requires further investigation. Moreover, through GO and GO tree analysis, we also identified some unregulated genes that were involved in viral transcription and viral life cycle, such as *RPS25* and *RPL10* [27, 28]. Because the HBV replication requires an obligatory reverse transcription step, the results imply that CD36 may modulate HBV transcription. For further exploration of CD36-specific genes, we established co-expression networks in the HepG2.2.15-CD36OE and HepG2.2.15-vector groups for DEGs according to the Pearson’s correlation of each pair of genes. Gene co-expression network analysis is a powerful method to explore the intrinsic organization of a transcriptome[29]. According to the co-expression networks of the DEGs, we identified the core genes accompanying CD36 overexpression. Among the “key regulatory” genes, we specially focused on the expression levels of *APOBEC3B*. A series of studies reported that *APOBEC3B* could edit HBV covalently closed circular DNA (cccDNA) and reduce HBV replication in vitro and in vivo [30]. In addition, Interferon-α treatment could upregulate the expression of *APOBEC3B* to induce cccDNA degradation [31]. Upon infection, the cccDNA serves as the template for HBV viral transcription and replication [32]. Point mutations inactivating *APOBEC3B* reduced the inhibitory activity on HBV replication to approximately 40% [33]. Our data demonstrated that CD36 overexpression could inhabit the expression of *APOBEC3B*, suggesting that CD36 overexpression may increase HBV replication by inhabited *APOBEC3B*.

In addition, we also identified many other genes that were significantly differentially regulated between the two cell lines involved in host metabolism. According to the KEGG pathway analysis, CD36 overexpression mainly affected the pathways of fat digestion and absorption, tryptophan metabolism, arachidonic acid metabolism, ribosomes, and gastric acid secretion. Furthermore, the apolipoprotein B (*APOB*) gene was significantly downregulated and phospholipase A2 group IIA (*PLA2G2A*) was significantly upregulated in the fat digestion and absorption pathway in the present study. A previous study suggested that CD36 could regulate the release of arachidonic acid (AA) from cellular membranes by activating cytoplasmic phospholipase A2, which contributes to the production of proinflammatory eicosanoids[34]. The downregulated genes oxoglutarate dehydrogenase-like (*OGDHL*) and dopa decarboxylase (*DDC*) were involved in tryptophan metabolism. A ribosome is an organelle that translates messenger RNA into a polypeptide chain in the course of protein synthesis. Ribosomal proteins play important roles in the regulation of transcription, DNA repair, cell proliferation and apoptosis. In the present study, some significant DEGs involved in ribosome pathways were upregulated. Thus, we inferred that the ribosome pathway is an important process that is affected by CD36. Previous studies reported that CD36 was involved in store-operated calcium flux [34]. Among the DEGs, we found that neurogranin (*NRGN*) was related to calcium signaling. Neurogranin is a member of the IQ motif class of calmodulin-binding proteins, which can be bound to the C-terminal domain of calmodulin to increase the Ca^2+^ dissociation rate [35]. Calcium signaling is a key factor in HBV and is involved in HBV DNA replication and HBV core assembly [36–39]. Therefore, CD36 may regulate the HBV life cycle through calcium signaling. In addition to calcium homeostasis, KEGG pathway analysis showed that the genes of potassium voltage-gated channel subfamily J member 10(*KCNJ10*) and potassium two pore domain channel subfamily K member 10(*KCNK10*) contributes to the GO terms of gastric acid secretion. *KCNJ10* gene encodes a member of the inward rectifier-type potassium channel family, which allows K^+^ flow into the cell [40]. *KCNK10* channel is an open rectifier which primarily passes outward current under physiological K^+^ concentrations [41]. In the present study, the *KCNJ10* was significantly upregulated and *KCNK10* was significantly downregulated, which could lead to increase cytoplasm concentration of potassium ion. Our results suggest that CD36 is involved in K^+^ homeostasis in the liver cells. Whether *KCNK10* and *KCNJ10* channel are participants in the HBV life cycle needs to be further investigation.

In conclusion, we reported, for the first time, that increasing the expression of CD36 could promote HBV replication in HepG2.2.15 cells. To understand the role of enhanced CD36 expression in host metabolism and the HBV life cycle in HBV replication cell lines, we performed RNA-seq between HepG2.2.15-CD36OE cells and HepG2.2.15-vector cells and identified 141 DEGs. The present study results provide significant clues for future studies and extend our knowledge regarding the synergistic effect of genes related to CD36 overexpression in an HBV replication background. Given the fact that CD36 increases HBV replication to a certain level, we speculate that for those HBV infected patients with a higher expression level of CD36, such as NAFLD and MS, proper control of CD36 or metabolism treatment may serve as an adjunctive therapeutic means which may have an additive effect to the conventional anti-viral drugs such as interferon and reverse-transcriptase inhibitors.

## Supporting Information

S1 Fig The expression of CD36 in HepG2 and HepG2.2.15 cells. (TIF)

S2 Fig Boxplot of the log transformed RPKM expression values across six transcripts. (TIF)

S1 Table Primers for quantitative real-time PCR. (DOC)

S2 Table Statistical results of quality control in the transcription group of all samples. (XLSX)

S3 Table Differentially expressed genes between HepG2.2.15-Vector cells and HepG2.2.15-CD36OE cells. (XLSX)
