# Supplementary figures and images for: Genome-Wide Transcriptome Analysis of CD36 Overexpression in HepG2.2.15 Cells to Explore Its Regulatory Role in Metabolism and the Hepatitis B Virus Life Cycle
Source: PLoS One. 2016 Oct 17;11(10):e0164787. doi: 10.1371/journal.pone.0164787 (PMC5066966; doi:10.1371/journal.pone.0164787)

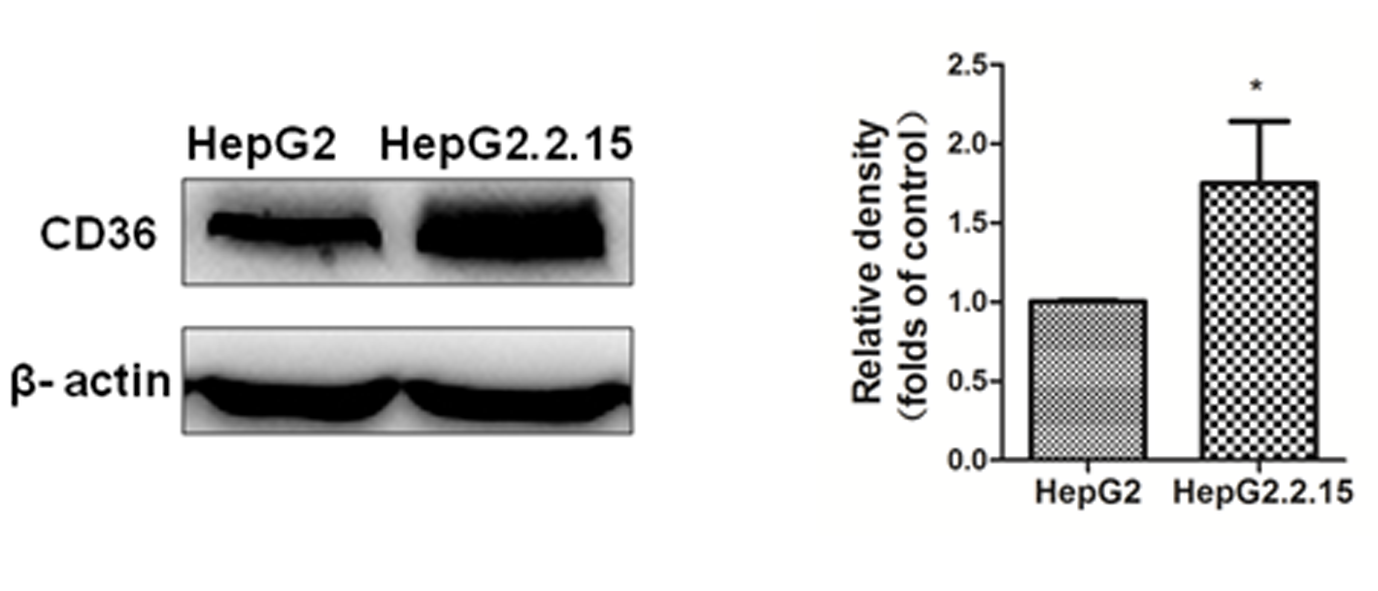

Supplement: S1 Fig — (TIF) [file pone.0164787.s001.tif]

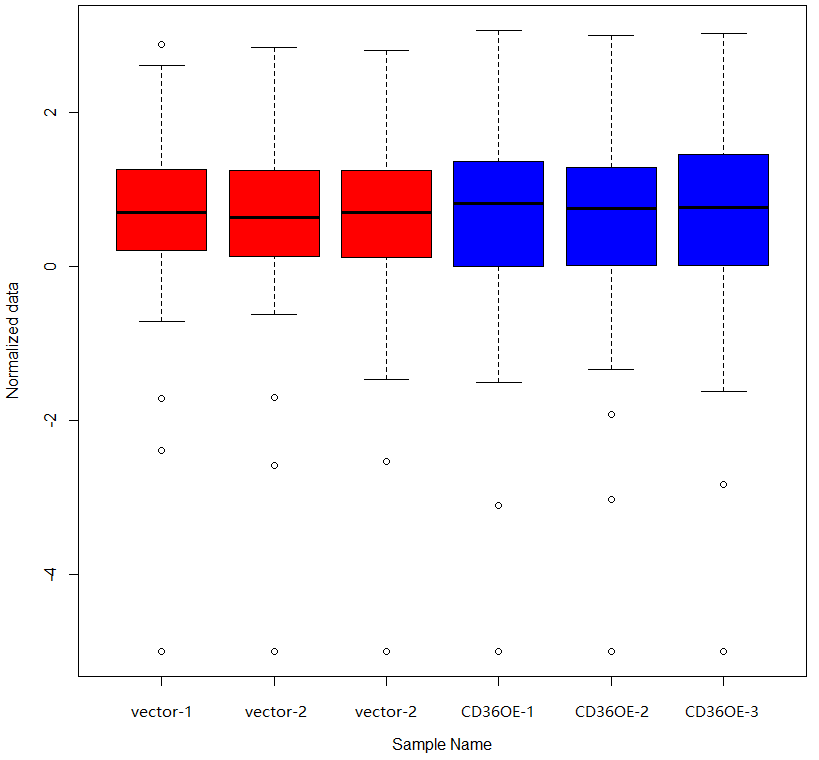

Supplement: S2 Fig — (TIF) [file pone.0164787.s002.tif]
