# Supplementary material for: Genome-Wide Transcriptome Analysis of CD36 Overexpression in HepG2.2.15 Cells to Explore Its Regulatory Role in Metabolism and the Hepatitis B Virus Life Cycle
Source: PLoS One. 2016 Oct 17;11(10):e0164787. doi: 10.1371/journal.pone.0164787 (PMC5066966; doi:10.1371/journal.pone.0164787)
Supplement: S1 Table — (DOC) [file pone.0164787.s003.doc]

**S1 Table Primers for quantitative real-time PCR**

| genes | primers | |
| --- | --- | --- |
| TotalHBV-DNA | Forward | 5’-TGCGGCGTTTTATCATATTCC -3’ |
|  | Reverse | 5’-ATACCTTGGTAGTCCAGAAGAACCA -3’ |
| CD36 | Forward | 5’- GAGAGAACTGTTATGGGGCTAT-3’ |
|  | Reverse | 5’-TTCAACTGGAGAGGCAAAGG -3’ |
| KCNK10 | Forward | 5’- CCCAAGAGCGCCACTAACG-3’ |
|  | Reverse | 5’- GACAAGGTAGACCACCACAAC-3’ |
| REG3A | Forward | 5’- AGCTACTCATACGTCTGGATTGG-3’ |
|  | Reverse | 5’- CACCTCAGAAATGCTGTGCTT-3’ |
| STC1 | Forward | 5’-CACGAGCTGACTTCAACAGGA -3’ |
|  | Reverse | 5’-GGATGTGCGTTTGATGTGGG -3’ |
| OGDHL | Forward | 5’-GGGCGTGGTATATGAGACCTT -3’ |
|  | Reverse | 5’- TGTGGTGAATCCAATCTGGTTG-3’ |
| RASSF2 | Forward | 5’-GCCACCAAACGTCCCTAGTC -3’ |
|  | Reverse | 5’-ACAAGTTGTAGGTCTTCAGATGC -3’ |
| PLA2G2A | Forward | 5’- ATGAAGACCCTCCTACTGTTGG-3’ |
|  | Reverse | 5’- GCTTCCTTTCCTGTCGTCAACT-3’ |
| NGRN | Forward | 5’- GCCTCTCCAGCTCTCTTGTT -3’ |
|  | Reverse | 5’-CATGCACACACGGAGATCAG -3’ |
| APOBEC3B | Forward | 5’-GACCCTTTGGTCCTTCGAC -3’ |
|  | Reverse | 5’- GCACAGCCCCAGGAGAAG-3’ |
| RPS28 | Forward | 5’-ACCCTTTTGGAGTCAGAGCG -3’ |
|  | Reverse | 5’-ATCTCAGTTACGTGTGGCGG -3’ |
| RPS25 | Forward | 5’-CGCACCCTGCAATATCTGAG -3’ |
|  | Reverse | 5’-TCCCCATGCGTGTTGTAATTC -3’ |
| APOB | Forward | 5’-TGAGGAGAAGAATCGAACCCT -3’ |
|  | Reverse | 5’- CTTGATTTCGTAGAGCAGACAGG-3’ |
| GPX2 | Forward | 5’- GAATGGGCAGAACGAGCATC-3’ |
|  | Reverse | 5’- CCGGCCCTATGAGGAACTTC-3’ |
| β-actin | Forward | 5’-CCTGGCACCCAGCACAAT -3’ |
|  | Reverse | 5’-GCCGATCCACACGGAGTA -3’ |
